# Motivations for enrollment in a COVID-19 ring-based post-exposure prophylaxis trial: qualitative examination of participant experiences

**DOI:** 10.1186/s12874-024-02394-0

**Published:** 2024-11-05

**Authors:** Julien Brisson, Rebecca Balasa, Andrea Bowra, David C. Hill, Aarti S. Doshi, Darrell H. S. Tan, Amaya Perez-Brumer

**Affiliations:** 1 Dalla Lana School of Public Health, University of Toronto, 155 College Street, 5th Floor, Room 554, Toronto, ON M5T 3M7 Canada; 2 Division of Infectious Diseases, St. Michael’s Hospital, Toronto, ON Canada; 3 Institute of Health Policy, Management, and Evaluation, University of Toronto, Toronto, ON Canada; 4 Department of Medicine, Temerty Faculty of Medicine, University of Toronto, Toronto, ON Canada; 5 MAP Centre for Urban Health Solutions, St. Michael’s Hospital, Toronto, ON Canada

**Keywords:** Canada, COVID-19, Experience, Research ethics, Research participation, Ring-based study, SARS-CoV-2

## Abstract

**Background:**

Ring-based studies are a novel research design commonly used for research involving infectious diseases: contacts of newly infected individuals form a ring that is targeted for interventions (e.g., vaccine, post-exposure prophylaxis). Given the novelty of the research design, it is critical to obtain feedback from participants on their experiences with ring-based studies to help with the development of future trials.

**Methods:**

In 2021, we conducted 26 semi-structured interviews with adult participants of a COVID-19 ring-based post-exposure prophylaxis trial based in Canada. We applied a purposive sampling approach and electronically recruited participants who tested positive for COVID-19 (Index Cases) and either agreed or declined for the study team to contact their potentially exposed contacts. We also included individuals who participated in the trial after being potentially exposed to an Index Case (known as Ring Members), and those who declined to participate after potential exposure. The methodological design of semi-structured interviews allowed participants to share their opinions and experiences in the trial (e.g., elements they enjoyed and disliked regarding their participation in the study).

**Results:**

The majority of participants in our study were women (62%) and the average age was 37.3 years (SD = 13.2). Overall, participants reported being highly satisfied with partaking in the ring-based trial. Notably, no substantial complaints were voiced about the trial’s design involving contact after exposure. The most common reason of satisfaction was the knowledge of potentially helping others by advancing knowledge for a greater cause (e.g., development of potential treatment to prevent SARS-CoV-2 infection). Other reasons were curiosity about participating in a trial, and an activity to fill free time during the pandemic. A central element of dislike was confusion about instructions with the trial (e.g., independent at home SARS-CoV-2 testing). Additionally, maintaining confidentiality was a crucial concern for participants, who sought assurance that their data would not be shared beyond the scope of the study.

**Conclusions:**

Our results have the potential to inform future research, including clinical trials such as ring-based studies, by incorporating insights from participants’ experiences into the development of study protocols. Despite some protocol-related challenges, participants expressed high satisfaction, driven by the desire to advance science and potentially aid others.

## Introduction

The history of ethically problematic research, exemplified by studies like the Tuskegee Syphilis Study [1] and the Stanford Prison Experiment [2], underscores the pivotal role of research ethics boards (REBs) in evaluating the ethical acceptability of research projects. However, an underexplored aspect of research ethics relates to the non-therapeutic benefits for participants, such as altruism in volunteering in a study [3]. This consideration gains prominence in unconventional research designs, such as network-based recruitment strategies and contact tracing for infectious diseases. In these scenarios, participants either recruit or are recruited by their close contacts, diverging from conventional recruitment methods like public advertisements or through healthcare professionals. These approaches may introduce a range of ethical challenges for REBs, including issues related to participant consent, confidentiality, and privacy. To better understand and address emerging ethical tensions when developing research protocols that rely on participant-based or informal recruitment, feedback from participants involved such studies must be considered [4].

Studying participants’ experiences in research projects enriches study protocols by aligning them with the perspectives of the population, thereby maximizing research benefits and upholding ethical integrity [5–7]. This is especially critical for innovative designs like ring-based studies, increasingly used for infectious disease research (e.g., vaccine, post-exposure prophylaxis [PEP] trials), where a complete set of contacts of newly exposed individuals are identified for prevention interventions [8–10]. The rationale for ring-based trial designs is two-fold; first, they allow for rapid identification of individuals at high risk of the outcome of interest; second, if the intervention works, they may limit onward transmission of the pathogen in the community. While ring-based studies hold public health potential, they introduce ethical complexities, especially in network-based recruitment involving an infectious agent. Collecting participant feedback is vital for understanding acceptability, feasibility, and ethical dimensions in study design. Consequently, the aim of our qualitative study was to explore participants’ experiences in participating in and supporting the recruitment for a ring-based PEP trial aimed at preventing SARS-CoV-2.

Our qualitative study focused on two key aspects of ring-based trials: participant motivations and trial experiences. The goal was to gather insights to guide the design of future infectious contact-based recruitment trials. This understanding is essential for addressing potential challenges in network-based recruitment, enhancing delivery of study materials, improving participant experiences, and increasing future research engagement [11].

## Methods

### Parent trial: CORIPREV-LR study

This paper presents findings from the experiences and perspectives of participants involved in the COVID-19 Ring-based Prevention Trial with Lopinavir/Ritonavir (CORIPREV-LR) study [12]. The CORIPREV-LR study was an open-label, multicenter, cluster-randomized trial evaluating the efficacy of a 14-day course of lopinavir/ritonavir (LPV/r) as PEP for preventing SARS-CoV-2 infection in high-risk exposed contacts of confirmed cases [13]. Clinical trial outcomes included: safety; symptomatic COVID-19; seropositivity; days of hospitalization attributable to COVID-19; respiratory failure requiring ventilatory support attributable to COVID-19; mortality attributable to COVID-19; psychological impact of COVID-19 exposure; and health-related quality of life). Data collection processes included detailed interviews, daily symptom/temperature diaries, electronic questionnaires, chart review, and laboratory measures (e.g., self-collected swabs for polymerase chain reaction tests; self-collected saliva samples; HIV self-test; blood samples for serology; and dried blood spots in a random subset of participants). The exclusion criteria were: (1) prohibited drug-drug interactions; (2) persons who are breastfeeding; and (3) persons currently using LPV/r. Full methods are published elsewhere [13].

The CORIPREV-LR trial was initiated at multiple sites across Canada, although recruitment activities only occurred in Toronto and Ottawa. The study team contacted individuals with a confirmed case of SARS-CoV-2 (i.e., Index Cases) following notice from the respective referral network (e.g., emergency rooms, testing centers, etc.). Index Cases were asked to provide the contact information of individuals deemed at high-risk of COVID-19 exposure due to their close contact with the respective Index Case. Among Index Cases who agreed to provide the contact information, informal consent was obtained from their close contact prior to relaying the contact information to the study team. The close contact (i.e., Ring Member) was then directly contacted by study staff in request for participation in the trial. Consenting Ring Members then received study materials and/or LPV/r through same-day courier services.

### Research design and data collection

We conducted a qualitative evaluation of the CORIPREV-LR trial using in-depth, semi-structured interviews with open-ended questions [14]. This research design’s flexibility allowed participants to share their thoughts and experiences with the trial [15]. Table 1 presents the main domains of the interview guide, which three research team members developed. The first section of the interview focused on demographics (e.g., age, employment). The following section explored COVID-19 experiences (e.g., understanding of public health messaging). The final section centered on participants’ experiences participating in the CORIPREV-LR trial (e.g., elements they enjoyed and disliked). Examples of questions in the interview guide that pertained to individual experiences with the trial were: “How did you hear about the CORIPREV-LR trial study?” “Can you please describe what helped you to participate in the CORIPREV-LR trial study?” “Can you please describe any challenges that you encountered while participating in the CORIPREV-LR trial study?”

**Table 1:** Interview Guide Main themes

Domains	Themes
1. Demographics	1.1 Social location
	1.2 Living situation
	1.3 School/Employment
	1.4 Social support
	1.5 Health background
2. COVID-19 experiences and potential risks	2.1 COVID-19 impacts
	2.2 Understandings of public health messaging and uptake
	2.3 Physical contacts (family, children, and employers)
3. Daily life amid COVID-19	3.1 Physical impacts of having COVID-19 (during and/or after)
	3.2 Psychosocial and mental impacts of having COVID-19 (during and/or after)
	3.3 Impacts of having COVID-19 on access to care
4. Experience with participation in the research study	4.1 Motivations for refusal or engagement
	4.2 Study participation
	4.3 Acceptability of contact tracing by study personnel
	4.4 Impacts of participation
5. Access to ongoing care	5.1 Current access to care
	5.2 Plans for future ongoing care

We conducted virtual qualitative interviews with 26 participants via Zoom from August to October 2021. All interviews were audio-recorded and were approximately 30–60 min in length. We did not evaluate the clinical efficacy or side effect profile of PEP as part of this qualitative evaluation. Researchers responsible for collecting qualitative data for this evaluation were not involved in the conceptualization nor implementation of the parent trial.

Three team members designed the semi-structured interview guide. They conducted interviews with one participant at a time, and led the analytic process (APB, AD, and RB). All interviewers had experience conducting interviews in previous health-based research projects and/or possess graduate-level training in qualitative research methods. To ensure methodological rigour, the semi-structured interview guide was informed by the parent study protocol. All participants were asked the same core questions related to motivations for enrollment and experiences with the trial. To mitigate concerns about confidentially and participant comfort to express their honest opinions and experiences, qualitative evaluation was conducted by interviewers not part of the parent CORIPREV-LR study team.

### Sampling and recruitment

**Fig. 1:**
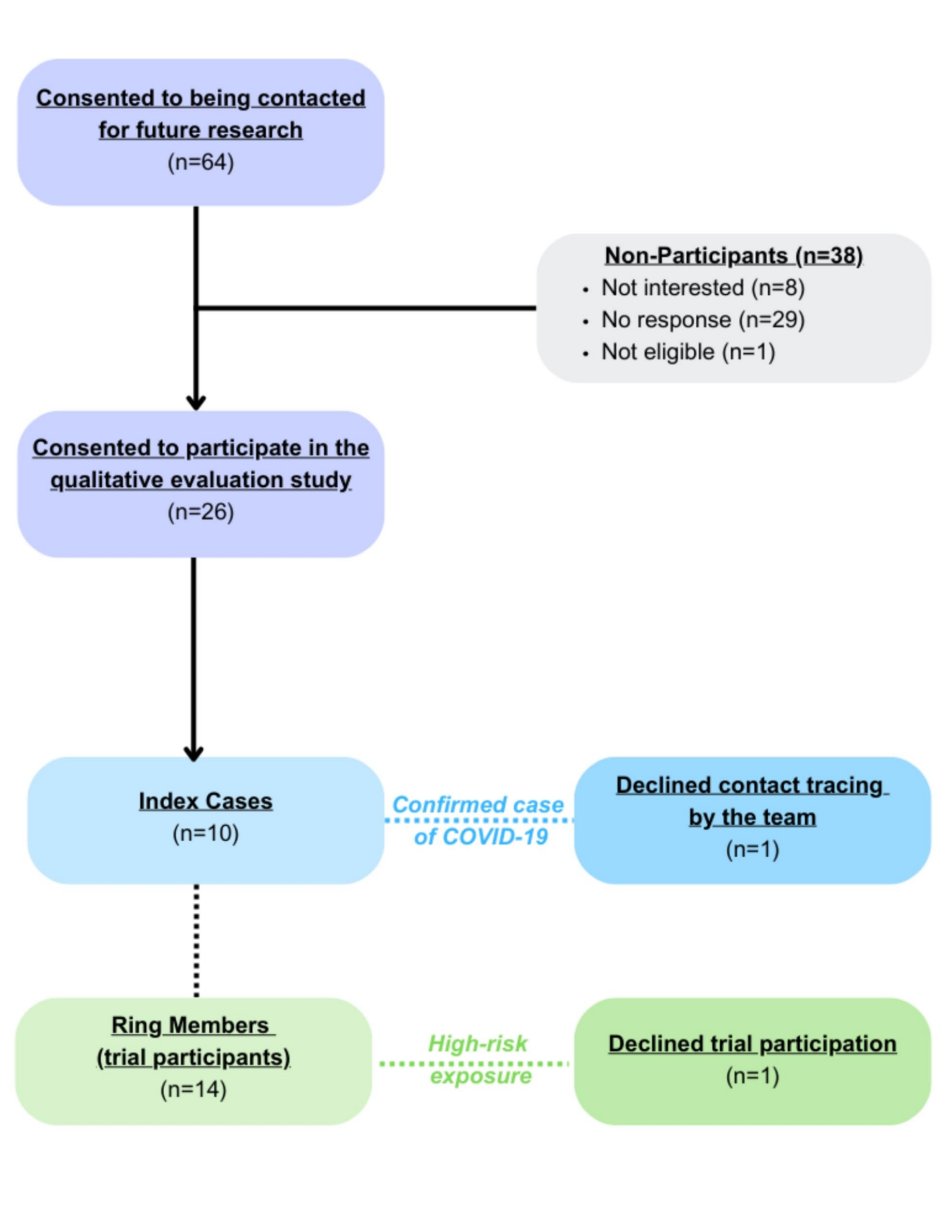
CORIPREV-LR Sample Types

We electronically recruited individuals directly involved with the CORIPREV-LR study who consented to be contacted for future research. The inclusion criteria required participants to be at least 18 years of age, speak English, demonstrate capacity to provide informed consent and fall into one of the following categories: (1) individuals with confirmed SARS-CoV-2 who did not allow the research team to contact their exposed contacts (Index Case – Declined); (2) individuals with confirmed SARS-CoV-2 who did allow the research team to contact their exposed contacts (Index Case); (3) high-risk exposed contacts of confirmed cases who declined participation in the trial (Declined Participation); and (4) high-risk exposed contacts of confirmed cases who participated in the trial (Ring Members) (Fig. 1). A purposive sampling approach [16] was applied to enhance the diversity and depth of perspectives within these participant categories. Exclusion criteria were: (1) prohibited drug-drug interactions; (2) persons who are breastfeeding; and (3) persons currently using LPV/r.

The qualitative evaluation sample size was guided by saturation [17] of themes related to evaluating participant experiences in the CORIPREV trial. To assess saturation, rapid and continuous preliminary analyses were conducted across study team during weekly meetings to ensure sufficient interviews were conducted.

### Data analysis

Semi-structured interviews were audio recorded and de-identified before being transcribed verbatim by a professional transcriptionist. The analytic process was guided by immersion crystallization [18, 19], which emphasizes examining data and reflecting on the experience of being immersed in the data analysis. The analysis was inductive and deductive to identify codes and relationships between codes in the iterative development of the codebook. A multi-step process was conducted via a web-based qualitative data management software, Dedoose (Version 9.0.17), to allow for a collaborative virtual workspace.

The coding was conducted in two stages. Initially, the semi-structured guide was used to inform the outline of domains within the codebook. Then, the core group of qualitative researchers that conducted interviews (APB, AD, and RB) independently reviewed the transcripts to assess other key domains, compare across themes, discuss, and reconcile any differences, and refine a set of codes for codebook and their definitions. A structured codebook was then developed transforming the themes into codes. The coding scheme was applied to an initial transcript by the two team members, and any discrepancies were discussed with a third member of the qualitative analysis group. AD and RB engaged in double coding to ensure consistency across approximately 25% of the transcripts [20] with changes tracked via an audit trail [21].

### Research ethics

#### Ethics approval

was received from the University of Toronto (Research Ethics Board #40710) and St. Michael’s Hospital (Clinical Trials Ontario Project ID #2126). Recruitment and participation were contingent on participants’ written informed consent with the knowledge of this study’s aims, risks, benefits, and voluntary participation. Participants were provided with a $40 CAD Amazon e-gift card to express gratitude for their participation. Recruitment and participation were contingent on participants’ written informed consent with the knowledge of this study’s aims, risks, benefits, and voluntary participation.

## Results

### Demographics

Of the 26 participants, most self-identified as women (62%), a third as men (31%), and less than a tenth as non-binary (8%) (Table 2). Almost three quarters identified as heterosexual (73%), and the remainder reported diverse sexual orientations. In terms of race/ethnicity, most participants self-identified as White (54%); the next most common response was South Asian (15%). The average age was 37.3 years (SD = 13.2), ranging from 19 to 71 years old. Two thirds of participants self-reported belonging in the “middle” and “upper middle” socioeconomic statuses (65%). It is important to note that demographic characteristics were determined based on individual responses to open-ended questions, without predefined criteria, allowing participants to self-identify without limitations. Most participants were high-risk exposed contacts of confirmed cases who participated in the trial (Ring Members; 54%) and individuals with a confirmed SARS-CoV-2 positive test who opted into the research team contacting their high-risk exposed contacts (Index Cases; 39%).

**Table 2:** Demographics of participants (*N* = 26)^1^

Characteristic	*N* (%)
Gender identity	
Women	16 (62)
Men	8 (31)
Non-binary	2 (8)
Sexual Identity	
Bisexual	2 (8)
Homosexual	2 (8)
Pansexual	1 (4)
Queer	1 (4)
Prefer not to answer	1 (4)
Heterosexual	19 (73)
Race/Ethnicity	
Black	2 (8)
Central Asian	1 (4)
East Asian	1 (4)
Latine	1 (4)
Middle Eastern	2 (8)
South Asian	5 (19)
White	14 (54)
Age	
<20	2 (8)
20–29	8 (31)
30–39	5 (19)
40–49	6 (23)
50–59	1 (4)
60+	4 (15)
Self-Reported Socioeconomic Status^2^	
Low	4 (15)
Middle	12 (46)
Upper Middle	5 (19)
High	4 (15)
Prefer not to answer	1 (4)
Sample Type	
Index Cases	10 (39)
Index cases - Declined	1 (4)
Ring Members	15 (54)
Declined Trial Participation	1 (4)

^1^Percentages were rounded to the nearest whole number due to the low participation rate

^2^These classifications were based on self-report, without predefined criteria

Experiences of study involvement varied between the four categories of interview participants. For example, engagement in the CORIPREV-LR trial, which involved potential risks in taking a pharmaceutical drug (e.g., undesirable side effects), was limited to Ring Members. To capture the multidimensional study design, our results are divided into two parts: (1) motivations and hesitancies with engaging in the CORIPREV-LR study; and (2) participants’ experiences with the CORIPREV-LR trial. Analysis is presented to capture the similarities and differences in both the experiences and perspectives of participants across the various sample types.

## Part 1: motivations and hesitancies with engaging in the CORIPREV-LR study

Amidst the COVID-19 pandemic and heightened clinical trial activities, we investigated the motivations behind participants’ decisions related to the CORIPREV-LR study.

### Altruism

Reinforced by the urgency produced by the COVID-19 pandemic, altruistic motivations underscored participants’ willingness to engage in the CORIPREV-LR study. Among Index Cases, altruistic motivations for research involvement were rooted in opportunities to transform the adverse outcome of testing positive for SARS-CoV-2 into “social participation in something that’s perceived as a public good” (59-year-old man, Index Case). Intertwined with perceptions of civic duty during the COVID-19 pandemic, Index Cases described perceiving importance in facilitating interactions between the research team and social networks deemed as high-risk exposures:

*Somebody called me asking me about this study and I said I would participate. Then I contacted my friend [to gauge interest] and she participated in the study and she did all this stuff. Very little is known about [COVID-19]*,* so I just wanted to help out in any way*,* shape*,* or form”* (31-year-old woman, Index Case).

Through facilitating these interactions, participants strongly endorsed the dual benefits of trial participation for science and society, highlighting how trial participation may “help move forward things for science, so we can better handle the pandemic and understand how it’s affecting people. Anything that helps move society forward” (41-year-old man, Index Case).

Ring Member participants, who were at high risk of contracting SARS-CoV-2 from their respective Index Case, saw their participation as a way to mitigate this risk. Many of these participants found value in the broader implications of taking a pharmaceutical drug “that is potentially beneficial for general society” (32-year-old woman, Ring Member). During a period when “there wasn’t any approved vaccine” and “nothing was available for people with COVID-19”, trial participation was described by these participants as a “win/win” situation: “There wasn’t really any long-term risk taking this drug for a short period of time, and there was the potential benefit. I thought, if this drug is already on the market, maybe it can be fast-tracked if it works” (44-year-old woman, Ring Member). Participants expressed hope in contributing to global efforts to combat the COVID-19 pandemic and generate findings that could potentially benefit COVID-19 patients worldwide:

*The COVID-19 pandemic is something that everyone in the world is now struggling with and even if I can help by a little bit*,* it would be nice to provide any sort of result or cure or even just medication to comfort some patients – anything would be better than nothing* (28-year-old woman, Ring Member).

Participants described a range of motivations for sharing data. Some participants expressed eagerness in “leaving their mark, however so small,” explaining how the CORIPREV-LR study “gave them somewhat of a voice” to share their experiences with COVID-19 through the provision of data (41-year-old man, Index Case). Paralleling these sentiments, one participant was particularly motivated to increase the representation of Black participants in COVID-19 clinical trials through their involvement:

*Especially being Black*,* my data adds to a dataset that is usually highly underrepresented when it comes to research studies. Across all the clinical trial studies*,* when it came to testing people outside of White populations*,* you can see their low numbers and it’s just like*,* ‘Oh wow*,* so is it safe for me?’ So*,* I would like to fill in that gap in clinical trial data* (40-year-old man, Index Case).

### “Something to Do”/Curiosity

The pandemic brought about various restrictive public health measures in Canada, including stay-at-home orders mandated in the province of Ontario. As a result, many people described experiencing boredom [22, 23], which motivated interest among those eligible for trial participation: “The CORIPREV-LR trial was a different activity to add to my schedule. It was a very small thing for me to do and it was already a period of time that was essentially blocked off because we were isolating” (32-year-old woman, Ring Member).

For some participants, the CORIPREV-LR trial was their first time participating in a clinical trial. Intrigued about what it entailed, the opportunity to participate in the trial component sparked curiosity among these participants, further incentivizing enrollment in the study: “I was really curious, I just thought it was a cool thing to do and it was really interesting to be part of, so I agreed to do it and didn’t really have any concerns” (44-year-old woman, Ring Member).

### Underlying hesitancies

Most participants in the qualitative evaluation study actively participated in the CORIPREV-LR study. However, two participants who declined CORIPREV-LR study participation disclosed underlying hesitancies that contributed to their decision. For instance, one participant with a confirmed SARS-CoV-2 infection declined to relay the contact information of their high-risk exposures due to fear of retribution from Public Health: “I guess I was just hesitant to participate in the study because I don’t know if I would get in trouble for having close contacts. I kind of wish I would have participated instead of being scared” (Index Case – Declined). Another participant, who declined trial participation, opted to wait for published study findings prior to taking PEP for COVID-19: “I was told the CORIPREV-LR study will give us, in the future, some results. We will see which is the correct way for the people’s life, for people’s environment, and for the science” (44-year-old woman, Declined Trial Participation).

While many Index Cases were eager to support the CORIPREV-LR study by relaying information to their high-risk exposures, few expressed uncertainties about whether they would engage as a Ring Member if roles were reversed. When asked about hypothetical interest in trial participation, some participants shared hesitancies surrounding how lopinavir/ritonavir may impact other existing health conditions. For instance, one participant who gave birth prior to study involvement disclosed the following: “I was still breastfeeding, so no I don’t think so. At some point, when I would not be breastfeeding, I might have considered it” (35-year-old woman, Index Case). Another participant also believed that they would have declined trial participation due to experiencing fatigue as a symptom of their SARS-CoV-2 infection: “I didn’t have a lot of energy at that time, so maybe I would have said no. I was so tired at that time, I just wanted to sleep” (27-year-old man, Index Case).

Because the CORIPREV-LR study collected various forms of data, such as biological samples and survey responses, participants also sought reassurance regarding adherence to confidentiality. As illustrated by one participant: “If you ask me if you can use this data as much as you can, please do. As long as you are keeping my confidentiality, I don’t have any problem” (35-year-old woman, Ring Member). Expanding on how data should remain confidential, one participant stressed the following:

*The important thing for me is that data usage should be restricted only to the study. It should be strictly confidential for me and for any other study participants. I think with all health care data*,* you try to make sure that it’s as confidential as possible* (64-year-old man, Ring Member).

## Part 2: participants’ experiences with the CORIPREV-LR trial

In the second part, we outline the aspects that the participants found enjoyable and then delve into the elements that they were most apprehensive about or disliked about the trial.

### Trial’s administrative process

Participants overwhelmingly described the CORIPREV-LR trial as “incredibly simple” (44-year-old woman, Ring Member) and “very time-efficient” (45-year-old woman, Ring Member). Participants felt “super engaged” and “really supported” (23-year-old woman, Ring Member) by the study staff while enrolled in the trial. As part of the trial, participants had to do self-swabbing tests and coordinate with the study team to send their samples to the laboratory. One participant reported: “Everything was sent and picked up by courier. We just had to leave it in the mailbox, so it couldn’t have been more convenient. The Zoom calls were once a week for three weeks only, so it really wasn’t that invasive” (71-year-old woman, Ring Member). Another participant commended the study for being highly organized and well thought out: “I think someone clearly thought very hard about how to deliver it in the most efficient and straightforward manner, so I don’t really have any suggestions for improvement” (32-year-old woman, Ring Member).

### Obtaining material and Informational resources

In addition to positive experiences associated with completing the research tasks of the trial, participants reported that receiving multiple at-home swabs to test for SARS-CoV-2 was a “big benefit” to them (64-year-old man, Ring Member), especially considering that the trial was conducted before the availability of SARS-CoV-2 rapid tests. Participants stated that “there were lots of swabs” and felt reassured “to have those negative results throughout the trial” (45-year-old woman, Ring Member). This access to material resources was described as “an added side effect to have extra tests done without leaving the house” (32-year-old woman, Ring Member) and “not having to go into the hospital to get tested for COVID” (40-year-old man, Index Case). Participants also expressed enjoyment in having consistent and updated results of SARS-CoV-2 status:

*Because we got tested at regular intervals*,* we were able to see like how long we remained SARS-CoV-2 positive. While I continue to be positive throughout the whole duration of the testing*,* my son was negative – like literally towards the end*,* so it’s interesting to see how the virus varies across people* (64-year-old man, Ring Member).

Amid the pandemic’s isolation, participants also reported the benefits of having access to informational support through health professionals within the study team. Reflecting on their partner’s participation in the CORIPREV-LR trial, one participant expressed appreciation for “having someone like a health professional to talk to” while navigating the uncertainties of living with a partner who tested positive for SARS-CoV-2 (24-year-old non-binary person, Index Case). In parallel, another participant reported that “the people from the study that I did come into contact with were friendly, professional, and informative” (28-year-old non-binary person, Ring Member). Connecting with the Research Coordinator who “was amazing” and “so accommodating” (27-year-old woman, Ring Member) was also a positive weekly connection for some participants:

*It was nice to do a Zoom check-in with study personnel. We’d be like*,* “oh*,* nice*,* see you the same time*,* the same place next week.” It was actually like a little extra community I had when I was sick which was really nice* (23-year-old woman, Ring Member).

### Challenges with Protocol requirements

Despite the ease and benefits of participating in the CORIPREV-LR trial, participants also described challenges for themselves and shared perceived potential barriers to participation for others. Particularly, participants who lived in apartment buildings raised concerns about potential SARS-CoV-2 transmission when transporting study materials:

*So*,* the first time we had to actually go downstairs*,* and I was not happy about that. But because both of us were*,* technically*,* I would say*,* positive for COVID-19. I didn’t consider it good infection control practice to go down and collect it*,* to give it to them in the car. So*,* I think I discussed that with the Research Coordinator*,* and she kind of changed that* (64-year-old man, Ring Member).

Some participants reported that needing access to an internet connection and device for Zoom check-ins, and the needed time to complete study tasks, could represent important barriers for some participants:

*So*,* you need to have a device with a camera to participate in the trial. The teaching sessions on how to do the swabs were over Zoom. Some of our chats were over Zoom. Things were dropped off to my door – Obviously someone that doesn’t have a home*,* or is in a shelter*,* wouldn’t be able to take part in this* (45-year-old woman, Ring Member).

In tandem, certain participants encountered challenges with some tasks involved with the trial: “Participation in the CORIPREV-LR trial impacted my day-to-day life a little bit. I had to remember to check my temperature, take the pill, keep track of when I have to do the next test, and take those surveys every day about my symptoms” (28-year-old woman, Ring Member). Another participant encountered challenges related to difficulty in conducting the at-home specimen collection, such as “having some issues doing some of the blood work tests” (27-year-old woman, Ring Member), and miscommunication on the limitations that trial participation may impose on other COVID-19 prevention strategies:

*I got my first vaccine dose late because I was under the impression that I shouldn’t get my dose while on the study. But then I just randomly asked at one of the weekly sort of interview check-ins. I said*,* “can I get vaccinated?” and then they were like*,* “oh my god*,* yes please*,* go get it whenever.” So*,* to be honest*,* like if I had asked sooner I probably would have been vaccinated sooner but because I didn’t ask and just assumed*,* I did end up getting my vaccine a little bit late compared to my social circles* (27-year-old woman, Ring Member).

## Discussion

To our knowledge, this is the first study to explore participants’ experiences in a ring-based prevention study on infectious diseases. Given the novelty of this study design, our findings contribute valuable insights into participant experiences with ring-based studies. These insights are crucial for refining future methodologies in similar contexts and help REBs and researchers understand participants’ perspectives and experiences [24].

Our study delves into the motivations and experiences of participants in the CORIPREV-LR ring-based prevention trial, shedding light on the broader benefits beyond potential pharmaceutical remedies. These findings offer valuable insights for designing future contact tracing trials, highlighting altruism as a key motivator. Altruism as a factor for engaging in health research has been documented in other studies [25–27]. For instance, individuals may participate to advance knowledge for a family member suffering from understudied illnesses or to contribute to the greater good by aiding in the development of treatments for diseases affecting their community or society at large. Future trials should emphasize societal benefits to boost enrollment. Previous studies have shown that financial incentive was a motivation for participants to enroll in research [28, 29], however, in our study, no participant expressed this motivation.

Participants’ data privacy concerns underscore the need for robust safeguards and clear communication. Addressing challenges like internet access and time commitments calls for flexible trial designs. This may involve offering alternative methods for tasks, ensuring accessibility, and streamlining administrative procedures to reduce participant burden. Incorporating these insights aligns with participant perspectives, improving ethics and effectiveness in contact tracing studies. Limited COVID-19 trial literature suggests similar participant motivations: contributing to the greater good, self and community protection, and improved healthcare access [30, 31].

Our findings suggest that future trials – such as contact tracing trials – can be designed to increase participants’ positive experiences by emphasizing the social benefits of participating, such as altruism and representation of minority groups. Additionally, the administrative process should be streamlined and efficient, with supportive study staff and ample resources provided to participants. Researchers should also consider the potential anxiety and burden that participants may experience when implementing strict study protocols, such as frequent follow-up tests and medication regimens.

The lack of research on participants’ experiences in clinical trials underscores missed opportunities to learn about how to improve study design. A recent scoping review explored the limited literature on participant experience and found that, overall, participants reported high levels of satisfaction with the trials and expressed a willingness to participate in future trials [8]. Gathering feedback from research participants regarding their trial experiences is crucial for ethically informed research protocols and increasing intervention effectiveness (e.g., reducing the risk of participant dropouts) [32]. Clinical trials are often associated with notions of risk and harm, making it essential to consider the voices of participants on aspects they might have enjoyed in participating in a clinical trial to provide perspectives.

One unique characteristic of ring-based studies is that participants are recruited based on their close contact with confirmed cases of infection. This design differs significantly from conventional recruitment methods, which often rely on self-referral (e.g., in response to recruitment posters), or referral by clinicians (e.g., based on knowledge of a candidate’s health status). It is noteworthy that participants did not express concerns or negative feedback about this peer-based recruitment strategies during their interviews. However, it is crucial to acknowledge the potential for selection bias inherent in this recruitment approach. Individuals who might feel uncomfortable or have reservations about participating in clinical research are less likely to volunteer for a ring-based study. Consequently, the study’s findings may predominantly reflect the experiences of those who are more amenable to participation and who may not represent the broader population. This self-selection bias can lead to an underrepresentation of individuals with negative perceptions or concerns about the recruitment process.

REBs are responsible for evaluating research projects to ensure participant protection. Research designs are generally categorized along a risk spectrum; for instance, semi-structured interviews are usually considered low risk, while randomized clinical trials are categorized as high risk. Faced with innovative methodologies like ring-based studies in infectious diseases, REBs may demonstrate heightened caution and hesitancy in approving these projects due to a lack of established precedent, particularly as it relates to network recruitment. However, conducting research with participants from previous ring-based studies on infectious diseases can furnish REBs with valuable insights. Such engagement not only enhances REBs understanding of these methodologies but also leverages participants’ experiences to improve the evaluation and guidance of future research in similar areas. Therefore, our study, which presents findings from the mostly positive experiences and perspectives of participants in a ring-based study on an infectious disease, can help future researchers and REBs in developing more informed, effective, and ethically sound research protocols.

### Limitations

This study has limitations, including selection bias and participant representation bias due to a small sample size, primarily consisting of white and/or cisgender women with access to and literacy with technology. Consequently, findings may not apply to the broader population. Future research should aim to diversify the sample across various social characteristics (e.g., race, gender, sexual orientation, age, socioeconomic status, and geographical locations) to enhance generalizability.

Also, the CORIPREV-LR trial investigated a drug with prior Health Canada approval for HIV treatment, enabling participants to seek safety information before providing consent. Trials with novel, experimental drugs may face different challenges due to higher uncertainty. Furthermore, selection bias might be present, as individuals who had negative experiences or declined participation in the main trial may be less inclined to participate in study extensions, potentially biasing our results towards a more favourable assessment. For example, anecdotal observations from the CORIPREV-LR research team [33] revealed that a number of individuals withdrew participation after receiving and opening their study kits, possibly due to feeling overwhelmed by the contents, such as plastic bags marked with biohazard symbols.

## Conclusion

The scarcity of research examining participant experiences in ring-based studies underscores the need for a deeper understanding of participants’ perspectives regarding this unique study design and distinct recruitment approach, to inform future trial design. Additionally, this enhances the capacity of research ethics boards to evaluate this novel approach, ensuring ethical conduct. Although limited by a small sample size, this study initiates the exploration of participant experiences in ring-based studies, resonating with previous literature emphasizing altruism as a key motivator. Future research should expand geographically and contextually, transcending the peak of the COVID-19 pandemic.

## Data Availability

The datasets generated and/or analysed during the current study are not publicly available due to respect for participants confidentiality, but are available from the corresponding author on reasonable request.
